# Proposed Clinical Algorithm for Pleuroparenchymal Fibroelastosis (PPFE)

**DOI:** 10.3390/jcm13133675

**Published:** 2024-06-24

**Authors:** Hideaki Yamakawa, Tsuneyuki Oda, Keishi Sugino, Takashi Hirama, Masamichi Komatsu, Takuma Katano, Taiki Fukuda, Tamiko Takemura, Yoshiaki Kubota, Tomoo Kishaba, Yasuhiro Norisue, Jun Araya, Takashi Ogura

**Affiliations:** 1 Department of Respiratory Medicine, Saitama Red Cross Hospital, Saitama 330-8553, Japan; 2 Department of Respiratory Medicine, Tokyo Jikei University Hospital, Tokyo 105-8461, Japan; araya@jikei.ac.jp; 3 Department of Respiratory Medicine, Kanagawa Cardiovascular and Respiratory Center, Yokohama 236-0051, Japan; oda.0950g@kanagawa-pho.jp (T.O.); ogura@kanagawa-junko.jp (T.O.); 4 Department of Respiratory Medicine, Tsuboi Hospital, Fukushima 963-0197, Japan; ks115108@tsuboi-hp.or.jp; 5 Department of Thoracic Surgery, Institute of Development, Aging and Cancer, Tohoku University, Sendai 980-8575, Japan; takashi.hirama.b5@tohoku.ac.jp; 6 Division of Organ Transplantation, Tohoku University, Sendai 980-8574, Japan; 7 First Department of Internal Medicine, Shinshu University School of Medicine, Matsumoto 390-8621, Japan; mskomatsu@shinshu-u.ac.jp; 8 Department of Respiratory Medicine and Allergology, Aichi Medical University, Nagakute 480-1195, Japan; katano.takuma.843@mail.aichi-med-u.ac.jp; 9 Department of Radiology, The Jikei University School of Medicine, Tokyo 105-8461, Japan; taiki.fukuda@gmail.com; 10 Department of Pathology, Kanagawa Cardiovascular and Respiratory Center, Yokohama 236-0051, Japan; tamikobyori@gmail.com; 11 Department of Cardiovascular Medicine, Nippon Medical School, Tokyo 113-0022, Japan; ykubota@nms.ac.jp; 12 Department of Respiratory Medicine, Okinawa Chubu Hospital, Okinawa 904-2293, Japan; kishabatomoo@gmail.com; 13 Department of Emergency and Critical Care Medicine, Tokyo Bay Urayasu Ichikawa Medical Center, Chiba 279-0001, Japan; norisue.yasuhiro@gmail.com

**Keywords:** pleuroparenchymal fibroelastosis, interstitial lung disease, clinical algorithm

## Abstract

Pleuroparenchymal fibroelastosis (PPFE) is characterized by fibrosis involving the pleura and subpleural lung parenchyma, predominantly in the upper lobes. As PPFE appears to occur in patients with heterogeneous etiologies, the disease course is thus also heterogenous, with some patients showing rapid progression while others have slow progression. Therefore, it is very difficult to predict prognosis with PPFE. Needless to say, this problematic matter has influenced the treatment strategy of PPFE patients. In fact, until now no evidence has been shown for use in creating an appropriate management algorithm for PPFE. We speculate that “uncoordinated breathing” is the most important reason for dyspnea in PPFE patients. Because monitoring of physique and not just pulmonary function and radiological evaluation is also very important, particularly in PPFE patients, this review focused on the characteristics of PPFE through an overview of previous studies in this field, and we proposed an algorithm as precision medicine based on the current evidence. Multiple views by the pulmonologist are needed to standardize a clinical algorithm that is necessary to correctly assess PPFE patients under the premise of maintenance of physique by providing appropriate nutritional care and pulmonary rehabilitation.

## 1. Introduction

Pleuroparenchymal fibroelastosis (PPFE) is a rare lung disease with nearly unique clinical features that was first identified by Amitani et al. as idiopathic upper-lobe fibrosis [1,2]. PPFE is a condition characterized by pleural fibrosis and subjacent parenchymal fibroelastosis predominantly involving the upper lobes [3,4,5]. Idiopathic PPFE (iPPFE) was included in the rare idiopathic interstitial pneumonias (IIPs) in the update of the international multidisciplinary classification of IIPs published in 2013 [6]. Defining the onset time of PPFE is an important determinant for the prognosis of the disease because iPPFE may have a long symptom-free subclinical stage in which lesions are confined at the apex of the lungs [3]. Another important point, which is even more essential than defining the onset of the disease, is determining the type of disease present [3]. There are some patients in whom PPFE involves only the bilateral upper lobes for a long time and does not invade adjacent lobes or lower lobes [1,3,4], and their prognosis is better than that of patients with multiple lobe involvement, especially with lesions involving the lower lobes such as in usual interstitial pneumonia (UIP) [3,7,8]. From the knowledge available at present, PPFE appears to occur in patients with heterogeneous etiologies, thus making the disease course also heterogenous [2,3,9,10,11,12]. Needless to say, this problematic matter has influenced the treatment strategy of PPFE patients. The aim of present review was to assess the characteristics of PPFE through an overview of previous studies in this field and then focus on the future treatment of PPFE by proposing an algorithm for the management of patients with PPFE.

## 2. Clinical Features

**Figure 1:**
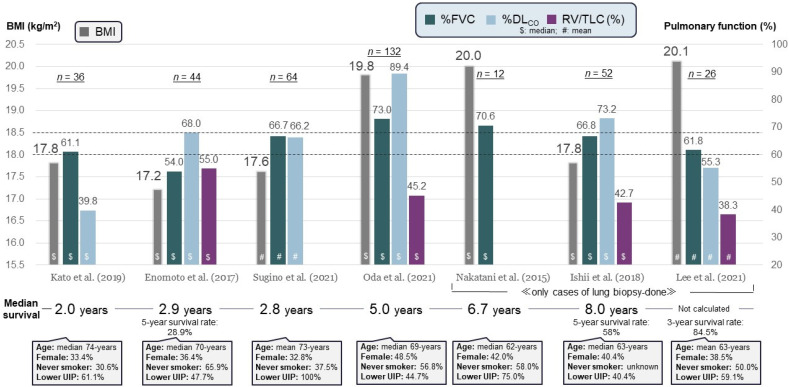
Summary of clinical characteristics reported by seven different studies [7,8,13,14,15,16,17] of patients with idiopathic pleuroparenchymal fibroelastosis (PPFE). BMI, body mass index; DL_CO_, diffusing capacity for carbon monoxide; FVC, forced vital capacity; RV, residual volume; TLC, total lung capacity; lower UIP, lower-lobe usual interstitial pneumonia.

Most patients present between 40 and 70 years of age, but PPFE has also been reported in children and those over 70 [2,9]. Previous studies reported a mean or median age in the 60s to 70s, with the ratio of females ranging from 30% to nearly 50% (Figure 1) [7,8,13,14,15,16,17]. PPFE at an advanced stage presents with symptoms such as dyspnea on exertion, cough, basolateral chest pain, and weight loss [11,13]. Platythorax, or flat chest, which is anteroposterior flattening of the thorax, is present in nearly half of the patients [5,11,13,18,19]. A flat chest, which may result from a congenital anomaly or may be a secondary change in the thorax due to shrinkage of the upper lung lobes through the long process of fibrosis, may inhibit lung compliance and blood flow in the upper lobes, leading to a ventilation–perfusion imbalance [19]. This may also result in a decreased range of motion of the rib cage.

Chest radiography in PPFE shows irregularly thickened apical areas, and reticular opacities appear in the bilateral upper lung fields [1,2,3,4,5,9,11,12,18,19]. Later, an upward shift of hilar structures is a characteristic finding on chest radiographs in PPFE [18]. Computed tomography (CT) shows dense pleural and subpleural consolidation coexisting with a reticular pattern, architectural distortion, and traction bronchiectasis, predominantly in the upper lobes [4,11]. PPFE may be accompanied by other patterns of interstitial lung disease (ILD) in the lower lobes. Previous cohort studies noted that more than half of patients with PPFE have lower-lobe ILD, especially of the UIP pattern (Figure 1) [4,5,7,8,9,11,12,13,14,15,16,17,18,19]. The lower-lobe involvement may occur with a reduction in volume and lifting of the diaphragm, which decreases its movement [19].

The most frequent histological finding in PPFE is a dense, band-like subpleural fibroelastosis that contains collapsed alveoli and runs parallel to the visceral pleura, which is itself partially thickened by hyalinized collagen. Higher-magnification views show that the alveolar lumens are filled with mature collagen associated with septal elastosis [5,19].

Pulmonary function testing shows decreased forced vital capacity (FVC) and total lung capacity (TLC), but the ratio of forced expiratory volume in 1 s (FEV_1_) to FVC is increased [3,5,9,16,18,19]. Fibrotic collapse of the upper lobes leads to compensatory overinflation of the lower lobes, resulting in an increased ratio of residual volume (RV) to TLC in PPFE [5,10,16,19,20,21]. Worsened gas exchange also appears as a restrictive impairment [19]. The diffusing capacity of carbon monoxide (DLco) is also decreased [7,8,13,16,17]. However, DL_CO_ is relatively preserved compared to FVC [19]. Similarly, desaturation on exertion (e.g., the lowest SpO_2_ in a 6 min walk test) is less common, unlike with idiopathic pulmonary fibrosis (IPF) [19].

Arterial blood gas analysis in PPFE patients reveals a tendency for normal PaO_2_ and A-aDO_2_ and high PaCO_2_ levels [21,22]. These findings differ from those of other ILDs, which develop gas exchange abnormalities that result in decreased levels of PaO_2_ and PaCO_2_ and an increased level of A-aDO_2_ [21,22,23]. Normal PaO_2_ and A-aDO_2_ levels indicate that the parenchyma distant from the pleura and subpleural regions is preserved [21]. Unlike other ILDs, in which the lower lobes are predominantly affected, PPFE is characterized by impairment primarily in the upper lobes with lower-lung perfusion, resulting in a reduced likelihood of hypoxemia. Watanabe et al. mentioned that elevated PaCO_2_ in PPFE does not result from obstructive lung disease but rather from mechanical restriction due to subpleural involvement, and furthermore, patients with PPFE at an advanced stage tend to develop hypercapnic respiratory failure [21]. Thus, elevated PaCO_2_ may be caused by hypoventilation or extrapulmonary restriction [9].

## 3. Important Points in the Mechanism of Dyspnea in PPFE Patients

**Figure 2:**
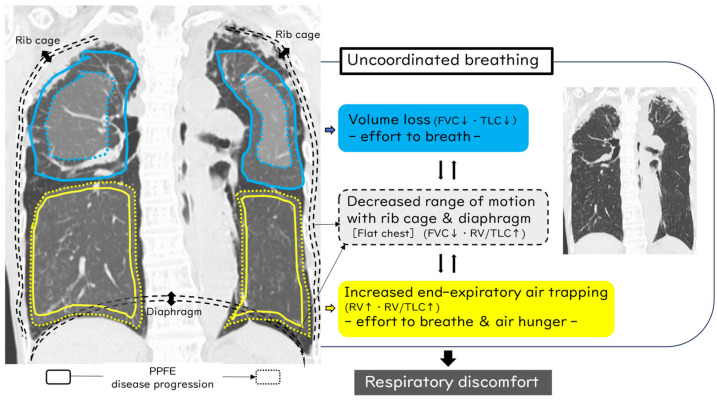
Mechanism of respiratory discomfort in patients with PPFE. Along with the progressive disease course, diminution of the upper lobes causes volume loss and decreased range of motion of the rib cage and diaphragm, which increases end-expiratory air trapping. Mainly, dyspnea due to dominant upper-lobe volume loss affects the “effort to breathe”, whereas air trapping and decreased range of motion of the rib cage and diaphragm lead to dyspnea experienced as “air hunger”. These factors often interact in diverse ways and may be the most important reasons for respiratory discomfort.

Considering the mechanism of dyspnea in PPFE patients based on clinical manifestations (flat chest), pulmonary function profile (decreased FVC and increased RV/TLC ratio), and arterial blood gas analysis (normal PaO_2_ and elevated PaCO_2_), we speculate that “uncoordinated breathing” is the most important reason for dyspnea in PPFE patients (Figure 2). Reduced TLC may be accompanied by mild to moderately increased RV [24]. Chua et al. mentioned this may include inhomogeneous lung emptying or increased end-expiratory air trapping [9]. Using three-dimensional (3D)-CT analysis, it was found that although the standardized upper-lobe volume in PPFE patients was less than half that of controls, importantly, the lower-lobe volume did not decrease [25]. Taken together, as shown in Figure 2, along with the progressive disease course, volume loss in the upper lobes (i.e., decreased FVC and TLC) in addition to decreased range of motion of the rib cage and diaphragm causes decreased FVC, increased RV/TLC, and limitation of expiration in other lobes, resulting in increased end-expiratory air trapping (i.e., further increased RV and RV/TLC), termed “traction air trapping”. Mainly, dyspnea due to dominant upper-lobe volume loss affects the “effort to breathe”, which results in increased respiratory muscle activity [26]. Contrastingly, air trapping and decreased range of motion of the rib cage and diaphragm lead to dyspnea experienced as “air hunger”, which is a primal homoeostatic warning signal of insufficient alveolar ventilation that can produce fear and anxiety, severely impacts the lives of patients, and is not limited to “effort to breathe” [26]. These factors often interact in diverse ways to cause uncoordinated breathing in individual PPFE patients and thus may relate to the most important reason for respiratory discomfort. A better understanding of the mechanisms of dyspnea may lead to the development of novel therapies and improved patient care [26].

## 4. Secondary PPFE

Several recent studies have explored and documented the possible etiologies of secondary PPFE, including radiation [27], bone marrow or stem cell transplantation [28,29], lung transplantation [28,30], connective tissue diseases (CTDs) [31,32,33], asbestosis [34], pneumoconiosis [34,35], metal lung [36,37], recurrent infections (e.g., pulmonary aspergillus [4] or *Mycobacterium avium* complex [38]), chemotherapeutic agents [39,40], and fibrotic hypersensitivity pneumonitis (fHP) [4,41,42]. Based on a report by Oda et al., fHP was relatively common in the secondary PPFE group [14]. Khiroya et al. speculated that PPFE in fHP might represent a progressive fibrosing immune-mediated response to an identified or unidentified inhaled antigen or allergen [41]. A systematic literature review focusing on CTD associated with PPFE found the most common PPFE-associated disease to be systemic sclerosis, followed by rheumatoid arthritis, inflammatory idiopathic myopathies, and other disease [43]. Several authors extended the understanding of the disease by highlighting non-idiopathic cases such as CTD and genetic causes [4,12,44]. Reddy et al. further suggested that PPFE of various etiologies may show varying patterns of disease progression and may also benefit from tailored disease management [4].

**Figure 3:**
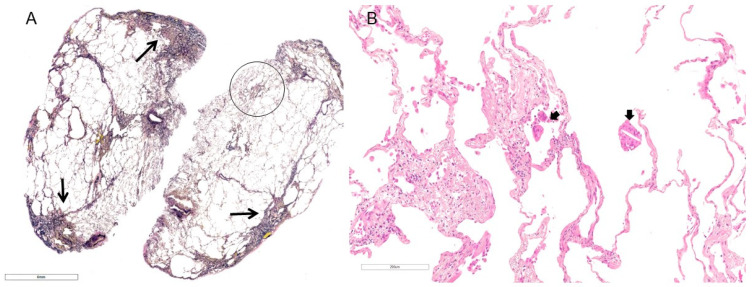
Surgical lung biopsy specimen from a patient with PPFE with fibrotic hypersensitivity pneumonitis. (**A**) High-magnification photomicrograph shows subpleural fibroelastosis (closed arrows) (Elastic van Gieson staining). (**B**) A high-power magnification view of the circled area in (**A**) shows mild centriacinar organization and fibrosis with multinucleated giant cells (thick arrows) (hematoxylin and eosin staining).

In the case of fHP as a secondary PPFE, although identification and elimination of the inciting antigen can be difficult to achieve in clinical practice, such management is most important to improving outcomes in these patients (Figure 3) [45]. Moreover, some patients with fHP may respond to anti-inflammatory agents, which may lead to a long-term benefit or slow the progression of pulmonary fibrosis, but there is little evidence for this [46]. As with CTD-ILD, autoimmune-mediated pulmonary inflammation is considered a key pathobiological pathway in these disorders, and immunosuppressive therapy is generally regarded as the cornerstone of treatment for severe and/or progressive CTD-ILD [47,48]. Therefore, when diagnosing PPFE, it is important first and repeatedly to rule out secondary PPFE, which may suggest additional therapeutic interventions, similarly with other IIPs, as described below, in the therapeutic management of PPFE.

## 5. Disease Behavior and Monitoring Markers

Ishii et al. noted that disease progression of PPFE is highly variable: some patients show rapid progression, whereas others have slow progression over 10–20 years following the initial clinical presentation [19]. Yoshida et al. suggested that the disease followed a heterogeneous clinical course because there appear to be two patterns of FVC decline: a rapid decline over a short period and a slow decline over a longer period [10]. The decline in FVC may not be linear, and they postulated that FVC declines gradually to a point at which it begins to decline rapidly [10]. It is clinically essential to monitor FVC to determine disease progression in patients with ILD [49]. However, it is difficult to perform pulmonary function tests on patients with advanced PPFE because of their severely impaired pulmonary function and increased risk for pneumothorax [50]. Therefore, a non-invasive and simple method of estimating the longitudinal decline in FVC would be highly important in the management of PPFE patients [51]. In other words, unlike with IPF, FVC is not an appropriate-enough surrogate marker for monitoring the disease course in PPFE [3,19].

Harada et al. explored the possibility that the flattened chest could be used to evaluate disease progression [52]. Ishii et al. also showed that such functional abnormalities seemed to be related to deformity of the chest cage, that is, as the thoracic cage becomes flattened, which impairs distension of the thoracic cage on inspiration, the FVC decreases but the RV/TLC ratio increases [19].

Because the annual % change in body weight was found to correlate with the annual change in FVC, changes in body weight may help estimate the decline in FVC in PPFE patients [51]. Decreased BMI was related to disease progression [16], and we also reported body weight loss to be a simple and useful prognostic indicator of nintedanib therapy in ILD patients [53]. Although FVC monitoring is important, non-invasive evaluation such as that of body weight changes can be a simple indicator for estimating disease progression or prognosis of PPFE, particular in the advanced stage [51].

Thus, to sum up, especially in PPFE patients, it is very important to monitor their physique (flat chest, body weight, or BMI) and not limit monitoring just to pulmonary function and radiological evaluations.

## 6. Prognostic Factors, Prognosis, and Causes of Death

Although there does not appear to be sufficient evidence, several previous studies showed that poor prognostic factors in PPFE patients include lower FVC and DL_CO_ values, deterioration in FVC during follow-up, hypercapnia (PaCO_2_ ≥ 50 Torr), elevated Krebs von den Lungen-6 (KL-6) (≥600 U/mL), standardized upper-lobe volume by 3D-CT (<30%), lower-lobe lung lesions (lower-lobe UIP), lower Geriatric Nutritional Risk Index (GNRI) (major malnutrition-related risk: GNRI < 82), decrease in GNRI during follow-up, body weight loss (≥−5%/year) during follow-up, development of pneumothorax, dyspnea grade ≥ 2 on the mMRC (Modified Medical Research Council) dyspnea scale, pulmonary hypertension, sleep-related hypoventilation, and higher peripheral neutrophil–lymphocyte ratio (NLR) (≥2.775) [7,8,14,23,25,50,51,54,55,56,57,58,59].

No large prospective studies of prognosis in PPFE patients are available [11]. As shown in Figure 1, median survival times are so diverse that no consensus has been reached. If we carefully consider several factors (i.e., BMI, FVC, %DL_CO_, %RV/TLC), these parameters (e.g., BMI < 18 kg/m^2^ and both %FVC < 70% and %DL_CO_ < 60%) may lead to useful predictive markers of poor prognosis indicating a survival time of <3 years [7,8,13,14,15,16,17]. As a cautionary point, the three right-most references [15,16,17] shown in Figure 1 included only cases of lung biopsy performed in patients with some physical reserve, and therefore, this point may relate to a better prognosis than that shown in other previous reports.

Median survival times of patients following subgroup analysis based on the presence or absence of poor prognostic factors were as follows: pneumothorax (1.5 vs. 2.8 years), PaCO_2_ ≥ 50 Torr (0.97 vs. 1.96 years), sleep-related hypoventilation (0.9 vs. not calculated), KL-6 ≥ 600 U/mL (2 vs. 5.1 years), lower-lobe UIP (1 vs. 5.2 years), pulmonary hypertension (1.3 vs. 4.2 years), and upper-lobe volume by 3D-CT < 30% (2.5 vs. 6.1 years) (Table 1) [7,16,23,25,50,58].

**Figure 4:**
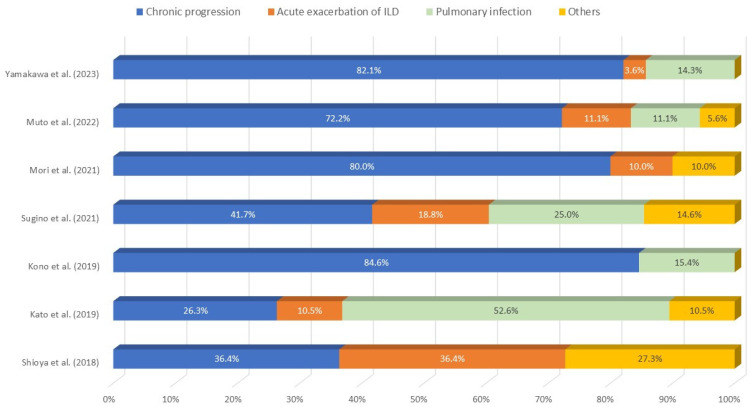
Causes of death reported by seven different studies [7,8,50,54,55,60,61] of patients with idiopathic PPFE. ILD, interstitial lung disease.

The three major causes of death were chronic progression (26.3–84.6%), acute exacerbation (AE) of ILD (3.6–36.4%), and pulmonary infection (0–52.6%) (Figure 4) [7,8,50,54,55,60,61].

## 7. Comorbidities

The three major comorbidities except for pulmonary hypertension in the patients with PPFE are reported to be pneumothorax, chronic pulmonary infection, and AE of ILD [7,8,19,50,54,55,60,61].

**Figure 5:**
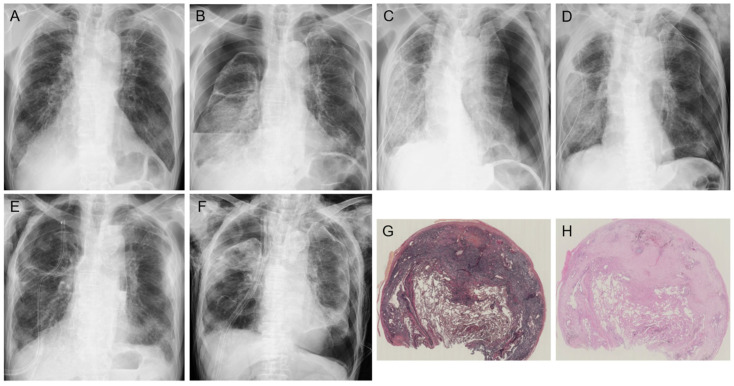
Disease course of a patient with refractory pneumothorax complicated by pleuroparenchymal fibroelastosis (PPFE). Chest X-ray at the diagnosis of idiopathic PPFE (**A**). After 4 months, chest X-ray showed bilateral pneumothorax (**B**). Immediately after insertion of a chest drain in the right intrathoracic space, a left-sided pneumothorax developed (**C**), which required insertion of a chest drain on the left side (**D**). However, persistent air leakage was noted in the right-sided drainage tube during repeated pleurodesis procedures (**E**), and the patient ultimately underwent surgical repair. After the surgical procedure managed with positive pressure ventilation, the patient’s bilateral pneumothorax worsened rather than improved (**F**). A lung specimen obtained during the surgical repair from near the suspected site of leakage showed a combination of visceral pleural fibrosis and intraalveolar fibrosis with elastosis ((**G**): Elastic van Gieson staining, (**H**): hematoxylin and eosin staining).

First, pneumothorax frequently develops during the clinical course of PPFE [3,33,50,61,62]. A subsequent case series of iPPFE showed that the incidence of pneumothorax after the diagnosis was 25–89% [50]. Kono et al. reported that the cumulative incidence rates of pneumothorax were 24.8%, 44.9%, and 53.9% at 1, 2, and 3 years, respectively [50]. A persistent air leak was observed in half of the patients who required chest drainage, and patients with pneumothorax had a significantly worse prognosis than those without pneumothorax [50]. Generally, pneumothorax with PPFE is likely to reoccur and be refractory to therapy (i.e., chest drainage, pleurodesis, surgical procedures) because of the high pressure required for lung expansion [50,63] (Figure 5). In addition, positive pressure ventilation, whether invasive or non-invasive, can often lead to ineffective lung expansion and may lead to higher risk of worsened pneumothorax because lung compliance itself is decreased in patients with PPFE owing to the flattened thoracic cage and subpleural fibrosis [23]. Although the mechanism of pneumothorax in patients with PPFE remains unclear, large cysts and multiple bullae in the apical fibrotic area may be associated with the high occurrence of pneumothorax, along with an altered resistance of the pleura to mechanical stress [3,19,33]. In addition, scarring of a visceral pleural defect following pneumothorax may lead to subpleural fibrosis [50,64]. In view of the pathological analysis between pneumothorax and ILD reported by Tachibana et al., pulmonary interstitial emphysema (PIE) begins with dissection of the interstitium in regions with fibrosis that then develops into a larger cystic space similar to bullous cysts in ILD [65]. Thus, some large PIEs reach the surface of the pleura and eventually develop air leaks, including pneumothorax and mediastinal emphysema [65]. We previously reported that para-carinal air cysts (PACs) were common (68%) in PPFE patients, and PACs were a significant risk factor for pneumothorax or pneumomediastinum [61]. Increased tracheal intraluminal pressure caused by chronic cough or obstructive ventilatory failure combined with weakened trachea-bronchus wall musculature from repeated respiratory infections can lead to the acquired form of PACs [66]. The high frequency of finding PACs may be compatible with compensatory increased end-expiratory air trapping of the lower lung against shrinking of the upper lung; therefore, compensatory hyperinflation of the lower lung may lead to additional pressure on the upper lobe and then to the creation of multiple air cysts along with PPFE lesions (Figure 2) [9,61]. We consider that pneumothorax, pneumomediastinum, and PACs in PPFE can be assumed to be caused by prominent negative intrathoracic pressure and consequently high transpulmonary and transluminal pressures due to the patient’s strong inspiratory effort to expand stiff lungs [67].

Hence, it has been suggested that pneumothorax can occur as a result of PPFE disease progression [50]. Needless to say, regardless of whether patients have severe ILD, having a lower BMI places them at higher risk of developing pneumothorax, and therefore, clinical management of physique is important to improve patient prognosis [68].

**Figure 6:**
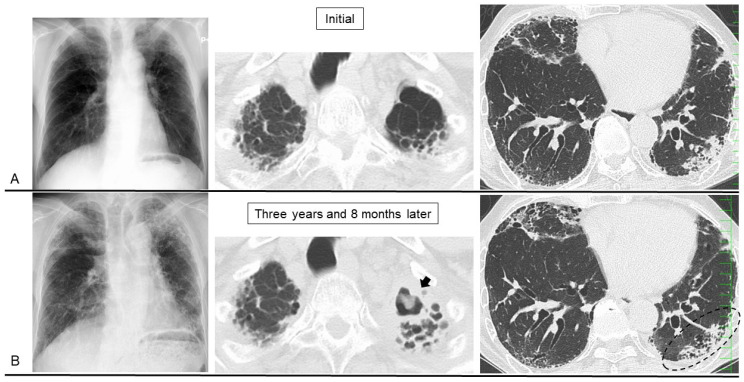
A patient with pleuroparenchymal fibroelastosis in whom the complication of pulmonary aspergillosis developed. ((**A**): **upper row**) The initial chest X-ray and computed tomography (CT) images showed pleural thickening, elevation of hilar opacities, and bullae in the upper lung fields. Consolidation with traction bronchiectasis was apparent in the lower lobe. ((**B**): **lower row**) Three years and 8 months later (following corticosteroid administration for 3 months), reticular and nodular opacities extended in the bilateral upper lung fields, and hilar opacities were further elevated. CT of the upper lung showed a fungus ball (thick arrow) at the location of the bullae, which was diagnosed as pulmonary aspergillosis. Despite this, the patient’s respiratory symptoms and consolidation shadows on CT partially improved (dashed ellipse) thanks to the corticosteroid.

Second, PPFE patients also suffer complications of chronic pulmonary infection caused by pathogens such as *Aspergillus* sp., nontuberculous mycobacteria (NTM), and *Pseudomonas aeruginosa* [61,69,70]. An association between *M. avium* complex, *Aspergillus* sp., and PPFE has been reported, and interstitial pneumonia as a comorbidity is associated with a worse prognosis [41]. *Pseudomonas aeruginosa* is also an opportunistic pathogen thought to infect patients with severe lung disease [71]. Obviously, prompt diagnosis and treatment are important to improve the prognosis. Although lung biopsy should be avoided in patients suspected of having PPFE because a postoperative iatrogenic pneumothorax commonly complicates the procedure, bronchoalveolar lavage and lung biopsy may be useful only when the possibility of combined pulmonary infection exists, such as by *Aspergillus* sp. (Figure 6), NTM, *P. aeruginosa*, or other pathogens. Obviously, the development of sarcopenia (skeletal muscle atrophy) as PPFE disease progresses is likely to cause dysphagia, which can lead to aspiration pneumonia.

**Figure 7:**
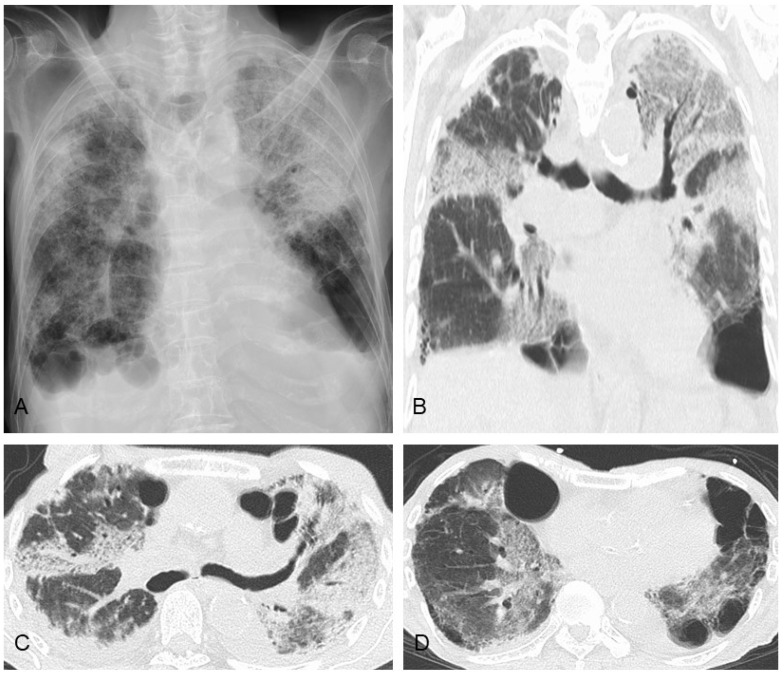
A patient with pleuroparenchymal fibroelastosis in whom acute exacerbation developed. (**A**) Chest X-ray. Bilateral consolidation with traction bronchiectasis and enlarged ground-glass opacities are shown on chest computed tomography (**B**–**D**). A small bilateral pleural effusion was also detected.

Third, AE of ILD (Figure 7) has been reported in PPFE patients, whether with or without lower-lobe UIP, and itself can be a direct cause of death [7,8,54,55,60,61,72,73]. However, AE tends to occur less often in patients with PPFE compared to those with IPF [74], and PPFE is associated with a lower number of deaths due to AE. Previous studies in IPF have shown that one of the risk factors of AE was low pulmonary function [75]; however, the incidence of AE was not high in PPFE patients with lowered pulmonary function [74]. Apparently, decreased pulmonary function did not seem to have affected the incidence of AE in these patients [74]. As there is no consensus on the mechanism and optimal management of AE in patients with PPFE, the treatment of AE in these patients is often performed in accordance with that of AE of IPF [75,76]. Thus, corticosteroids are often administered to patients with AE of IIP in daily practice [76]. However, to date, their efficacy has not been validated, and in addition, numerous side effects, including infections and pneumothorax, have been correlated with corticosteroids [69,77]. In the INBUILD trial, nintedanib as an anti-fibrotic agent significantly reduced AE or death in patients with progressive fibrosing ILD of various etiologies [78]. Because only a very few patients with PPFE were included in that study, further studies will be required to determine the optimal approach to AE in patients with PPFE.

## 8. Proposed Management Algorithm for PPFE

**Figure 8:**
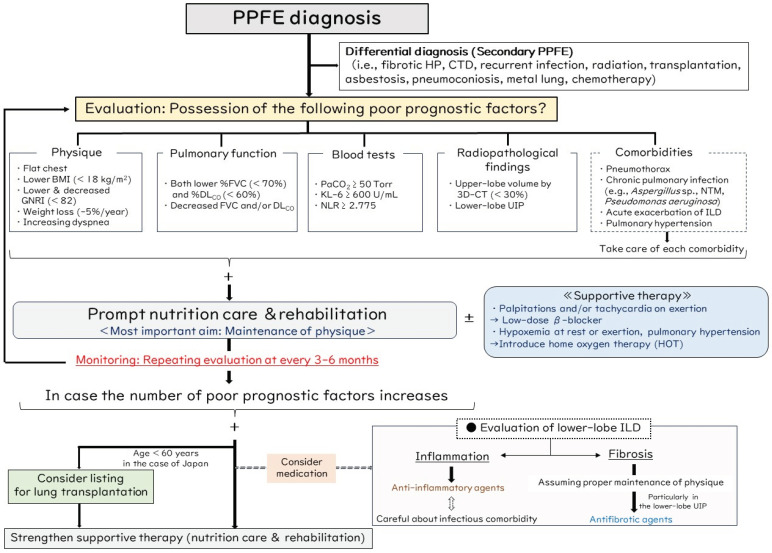
Proposed management algorithm for PPFE. PPFE, pleuroparenchymal fibroelastosis; HP, hypersensitivity pneumonitis; CTDs, connective tissue diseases; BMI, body mass index; GNRI, Geriatric Nutritional Risk Index; FVC, forced vital capacity; DL_CO_, diffusing capacity for carbon monoxide; KL-6, Krebs von den Lungen-6; NLR, neutrophil-lymphocyte ratio; 3D-CT, three-dimensional computed tomography; UIP, usual interstitial pneumonia; NTM, nontuberculous mycobacteria; ILD, interstitial lung disease.

No evidence has been shown to be appropriate for use in a management algorithm for PPFE. Limiting their study to candidates for lung transplantation in Japan, Shiiya et al. reported that the factors of a low BMI, short 6 min walking distance, hypoxemia, and an inability to perform the DL_CO_ test were not associated with poorer survival in the iPPFE group, although these were associated factors in other idiopathic ILDs [79]. In addition, some patients with iPPFE and these unfavorable factors survived longer than expected and eventually underwent successful lung transplantation, whereas almost all of the patients with other idiopathic ILD and these factors died while awaiting transplantation [79]. As a premise, these results suggest that the characteristics and prognostic factors of PPFE are distinct from those of other ILD; therefore, because it is very difficult to predict prognosis with PPFE, we have proposed the clinical algorithm shown in Figure 8.

After clinicians consider the possibility of the differential diagnosis as secondary PPFE, they should evaluate previously reported poor prognostic factors, as stated in Section 6, from multiple viewpoints, such as physique (increasing dyspnea, flat chest, low BMI < 18 kg/m^2^, low or decreased GNRI < 82, weight loss −5%/year), pulmonary function (lower %FVC < 70% and %DL_CO_ < 60%, decreased FVC and/or DL_CO_ during follow-up), blood tests (PaCO_2_ ≥ 50 Torr, KL-6 ≥ 600 U/mL, NLR ≥ 2.775), radiopathological findings (upper-lobe volume by 3D-CT < 30%, presence of lower-lobe UIP), and respiratory comorbidities (pneumothorax, chronic pulmonary infection, AE of ILD, pulmonary hypertension). If the patient has ever had of any of these factors, a prompt increase in nutritional input and pulmonary rehabilitation should be the interventions applied for maintenance of physique as the most important aim in treating PPFE. Pulmonary rehabilitation may be an efficacious non-pharmacological approach for managing disabilities in patients with PPFE [54]. As maintenance of physique can lead to the prevention of respiratory comorbidities, we speculate that change in body weight may be the most useful index when evaluating PPFE patients [51].

Thereafter, evaluation of the poor prognostic factors being monitored should be repeated every 3–6 months. If the number of poor prognostic factors increases, timing of the patient’s listing for lung transplantation should be considered. Note that an age limitation is enforced for recipients of lung transplantation in Japan, and patients must be under 60 years of age to be registered on the waiting list for lung transplantation. Although the proposal by an international guideline for timing the listing of ILD patients for lung transplantation is based on a decline in the FVC and/or DL_CO_, desaturation, exercise capacity, pulmonary hypertension, pneumothorax, and having a history of AE, to reiterate, the disease course of PPFE patients is not always the same as that of other ILD patients [79,80,81,82]. We think it important to evaluate patients from multiple viewpoints to be alert to the signs of a poor prognosis. In addition, given the difficulty in prognosticating PPFE and the variability in timing for patients listed for lung transplantation, it is crucial at this stage to refer patients with PPFE to transplant centers as promptly as possible.

When considering medication, we focus on the assessment of inflammatory or fibrotic changes in the high-resolution CT findings, particularly in lower-lobe ILD. Inflammatory change is assumed when ground-glass opacities and consolidation are present, whereas fibrotic change is assumed with reticulation with traction bronchiectasis and honeycombing [83]. An example is shown in Figure 6B, in which a patient had consolidation (dashed ellipse) in the lower lobe. Although chronic pulmonary aspergillosis had occurred, the respiratory symptom of persistent cough and the lower-lobe lesion partly improved after corticosteroid therapy. As in this case, prednisolone doses higher than 10 mg are associated with a high risk of an infectious comorbidity [84], so an immunosuppressive agent (e.g., cyclosporine, tacrolimus, mycophenolate mofetil, azathioprine) other than corticosteroid may be another useful option [43,85]. On the other hand, if we determine that fibrosis is dominant in lower-lobe ILD, we consider starting an antifibrotic agent such as nintedanib, which was shown to be effective in progressive fibrosing ILD of various etiologies [78]. Nintedanib is a potential treatment for patients with PPFE and lower-lobe UIP because fibrosis in this condition consists not only of elastic fibers but also collagen fibers, especially in the lower lobes [74]. Some patients treated with nintedanib showed significantly slower declines in FVC compared to patients without treatment [86], but another study showed no such effect of nintedanib [87]. Thus, the efficacy of nintedanib in patients with PPFE remains controversial. Importantly, we suggest that nintedanib administration be considered only if maintenance of good physique in the patient is assumed because lower weight tends to be associated with weight loss, which is a common side effect of nintedanib, and nintedanib itself (and not ILD alone) may accelerate weight loss as an adverse event, thus leading to its early discontinuation [53,88]. Because long-term persistent use of nintedanib may lead to a favorable prognosis, maintenance of good physique is of major importance when considering that decreased BMI is related to disease progression in patients with PPFE [16]. In addition to nintedanib, the effects and applications of other anti-fibrotic drugs such as pirfenidone need to be elucidated for the treatment of PPFE, particularly in the context of fibrosis in lower-lobe ILD.

As supportive therapy, interventions of home oxygen therapy (HOT) and a low-dose β-blocker may be one option in PPFE patients. Current guidelines recommend that patients with IPF and clinically significant resting hypoxemia and/or pulmonary hypertension due to pulmonary fibrosis should be treated with long-term HOT, although this is based on very low-quality evidence [89]. As an example, the clinical course and survival of PPFE patients who have been started on HOT are still not fully understood. Although a systematic review showed no consistent acute effects of short-term oxygen therapy on dyspnea during exertion in ILD, HOT may lead to increased exercise capacity by reducing breathlessness and increasing physical capacity through improved gas exchange [90]. Therefore, administration of HOT may be considered for PPFE patients with hypoxemia at rest or on exertion. In contrast, non-invasive positive pressure ventilation (NPPV) is sometimes administered when hypercapnia (elevated PaCO_2_) is present in patients with a chronic respiratory disease such as chronic obstructive pulmonary disease (COPD) and neuromuscular disease [23]. However, whether NPPV is effective in treating PPFE patients with hypercapnia is controversial [23,37]. One report showed that NPPV treatment tended to improve the prognosis in PPFE patients with sleep-related hypoventilation [37], whereas it was not effective for most other PPFE patients [23]. Unlike in COPD, lung compliance is usually decreased in patients with PPFE owing not only to a flattened thoracic cage but also to subpleural pulmonary fibrosis [21,38]. Thus, NPPV may be uncomfortable for PPFE patients with hypercapnia due to the need for increased high positive pressure to overcome the decreased lung compliance to obtain effective ventilation, which may increase the risk of developing pneumothorax. Therefore, we would not recommend NPPV treatment in most PPFE patients with hypercapnia, particular those with an advanced stage.

**Figure 9:**
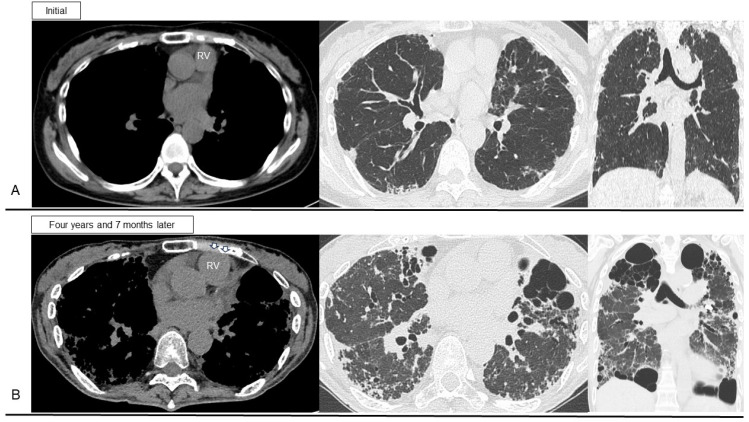
A patient with pleuroparenchymal fibroelastosis (PPFE) who experienced improvement of respiratory-induced symptoms such as palpitations and tachycardia on exertion following low-dose β-blocker treatment. (**A**) Initial diagnosis of PPFE. (**B**) At 4 years and 7 months later as the disease extent of PPFE further progressed, the patient complained of palpitations due to anatomical changes of thinning of the chest wall and compression of the right ventricle (RV) (arrows).

The chest wall abnormality of platythorax (flat chest) is a unique characteristic of PPFE [18,19,91]. In patients with pectus excavatum as a representative chest wall abnormality, the sternum may compress the lungs and heart, potentially leading to various symptoms, such as rapid heartbeat or heart palpitations, chest pain, and fatigue [92]. Progression of such a thoracic deformity may persist in compressing the right ventricle, thus causing structural and hemodynamic consequences that can lead to sinus, atrial, or ventricular tachycardia [93,94]. Increased right atrial pressure and dilation can also lead to increased sympathetic tone [95]. As an aside, obstruction of airflow and loss of lung elasticity can lead to lung hyperinflation that compresses the heart and elevates intrathoracic pressure, which reduces venous return and thus right ventricular output in patients with COPD [96]. Additionally, hyperinflation and greater changes in intrathoracic pressure during respiration might enhance ventricular preload and afterload, resulting in left ventricular dysfunction and heart failure [97]. Administration of β-blockers such as bisoprolol decreases the mortality rate through lowering of the heart rate and sympatholytic effects, so β-blockers might benefit patients with heart failure and COPD [98]. Considering PPFE, compression of the heart due to both chest wall abnormality (platythorax) and increased end-expiratory air trapping in the lower lobes may lead to increased oxygen consumption and sympathetic tone, as with severe emphysema in patients with COPD. In general, appropriate sinus tachycardias are managed primarily by addressing the underlying condition resulting in the fast rate [99]. In symptomatic (e.g., palpitations, chest pain) patients with inappropriate sinus tachycardia unresponsive to lifestyle modification strategies, β-blocker therapy may be helpful to slow the rate [100]. In practice, we sometimes use a low-dose β-blocker (bisoprolol) in those PPFE patients who complain of palpitations and/or tachycardia on exertion, and we have experienced improvement of these symptoms in some cases (example case: Figure 9). The PPFE patient losing body weight may complain of palpitations due to anatomical changes (air trapping in the lower lobe, thinning of the chest wall, right ventricular compression), and we consider a low-dose β-blocker a supportive option that may be effective in relieving symptoms and preventing secondary heart failure due to pulmonary disease, although there is no direct evidence for this.

Because PPFE is a progressive and highly symptomatic disease with a poor prognosis similar to IPF, it is important to improve patient quality of life through the appropriate use of opioids to relieve cough and dyspnea and to introduce advance care planning in the early stage of the disease [101].

## 9. Conclusions

Disease behavior of PPFE is highly variable, as its clinical course is heterogenous, with some patients showing rapid progression and others slow progression. Thus, predicting prognosis in PPFE is very difficult. Moreover, no evidence has been presented for use in an appropriate management algorithm for PPFE. Therefore, we should consider precision medicine as personalized medicine by monitoring patient physique in addition to pulmonary function and radiological evaluation. Above all, finding an effective treatment for PPFE remains a major clinical challenge [9]. Thus, a clinical algorithm needs to be standardized that incorporates multiple viewpoints of the pulmonologist to correctly approach the treatment of PPFE patients under the premise of maintenance of physique by providing appropriate nutrition care and pulmonary rehabilitation.

## Figures and Tables

**Table 1:** Median survival in each subgroup of patients with PPFE with (+) and without (−) poor prognostic factors as reported in previous studies.

Author (Year)	No. of Patients	BMI (kg/m^2^)	%FVC	%DL_CO_	Poor Prognostic Factor	Median Survival (Years)	
						+	−
Ishii et al. [56] (2018)	52	Median 17.8	Median 66.8	Median 73.2	KL-6 ≥ 600 U/mL	2	5.1
Kato et al. [7] (2019)	36	Mean 17.8	Mean 61.1	Mean 39.8	Lower-lobe UIP	1	5.2
Kono et al. [50] (2021)	89	Mean 17.0	Mean 61.9	Mean 84.9	Pneumothorax	1.5	2.8
Yabuuchi et al. [58] (2022)	52	Median 17.5	Median 52.2	Median 60.7	Sleep-related hypoventilation	0.9	NC
Muto et al. [55] (2022) *	83	Median 17.7	Median 62.2	Median 84.7	Pulmonary hypertension	1.3	4.2
Fukada et al. [25] (2022)	132	Mean 16.8	Mean 64.4	Mean 90.7	Standardized upper-lobe volume < 30%	2.5	6.1
Kinoshita et al. [22] (2023)	47	Mean 17.2	Mean 61.9	Mean 82.4	PaCO_2_ ≥ 50 Torr	0.97	1.96

* This study included patients with secondary PPFE and was not limited to those with idiopathic disease. PPFE, pleuroparenchymal fibroelastosis; BMI, body mass index; FVC, forced vital capacity; DL_CO_, diffusing capacity for carbon monoxide; NC, not calculated; KL-6, Krebs von den Lungen-6; UIP, usual interstitial pneumonia.
